# Widespread and persistent invasions of terrestrial habitats coincident with larval feeding behavior transitions during snail-killing fly evolution (Diptera: Sciomyzidae)

**DOI:** 10.1186/1471-2148-12-175

**Published:** 2012-09-10

**Authors:** Eric G Chapman, Andrey A Przhiboro, James D Harwood, Benjamin A Foote, Walter R Hoeh

**Affiliations:** 1 Department of Entomology, University of Kentucky, Lexington, KY, 40546, USA; 2 Zoological Institute, Russian Academy of Sciences, Universitetskaya nab. I St., Petersburg, 199034, Russia; 3 Evolutionary, Population, and Systematic Biology Group, Department of Biological Sciences, Cunningham Hall, Kent State University, Kent, OH, 44242, USA

## Abstract

**Background:**

Transitions in habitats and feeding behaviors were fundamental to the diversification of life on Earth. There is ongoing debate regarding the typical directionality of transitions between aquatic and terrestrial habitats and the mechanisms responsible for the preponderance of terrestrial to aquatic transitions. Snail-killing flies (Diptera: Sciomyzidae) represent an excellent model system to study such transitions because their larvae display a range of feeding behaviors, being predators, parasitoids or saprophages of a variety of mollusks in freshwater, shoreline and dry terrestrial habitats. The remarkable genus *Tetanocera* (Tetanocerini) occupies five larval feeding groups and all of the habitat types mentioned above. This study has four principal objectives: (*i*) construct a robust estimate of phylogeny for *Tetanocera* and Tetanocerini, (*ii*) estimate the evolutionary transitions in larval feeding behaviors and habitats, (*iii*) test the monophyly of feeding groups and (*iv*) identify mechanisms underlying sciomyzid habitat and feeding behavior evolution.

**Results:**

Bayesian inference and maximum likelihood analyses of molecular data provided strong support that the Sciomyzini, Tetanocerini and *Tetanocera* are monophyletic. However, the monophyly of many behavioral groupings was rejected via phylogenetic constraint analyses. We determined that (*i*) the ancestral sciomyzid lineage was terrestrial, (*ii*) there was a single terrestrial to aquatic habitat transition early in the evolution of the Tetanocerini and (*iii*) there were at least 10 independent aquatic to terrestrial habitat transitions and at least 15 feeding behavior transitions during tetanocerine phylogenesis. The ancestor of *Tetanocera* was aquatic with five lineages making independent transitions to terrestrial habitats and seven making independent transitions in feeding behaviors.

**Conclusions:**

The preponderance of aquatic to terrestrial transitions in sciomyzids goes against the trend generally observed across eukaryotes. Damp shoreline habitats are likely transitional where larvae can change habitat but still have similar prey available. Transitioning from aquatic to terrestrial habitats is likely easier than the reverse for sciomyzids because morphological characters associated with air-breathing while under the water's surface are lost rather than gained, and sciomyzids originated and diversified during a general drying period in Earth's history. Our results imply that any animal lineage having aquatic and terrestrial members, respiring the same way in both habitats and having the same type of food available in both habitats could show a similar pattern of multiple independent habitat transitions coincident with changes in behavioral and morphological traits.

## Background

Some of the most important evolutionary innovations in the history of life on Earth resulted from transitions between aquatic (freshwater) and terrestrial habitats. The colonization of land by unicellular aquatic plants [1,2] and their eventual transformation into vascular plants helped shape terrestrial environments and paved the way for the evolution of the majority of the eukaryotic species alive today. Other key lineage diversifications that occurred following transitions from aquatic to terrestrial habitats include those of tetrapod vertebrates [3], millipedes [4], scorpions [5], other arachnids [2,6,7], earth worms [8] and nematodes [9]. Whereas the ancestral insect originated in a terrestrial environment [10-12], insects are one of the most successful colonizers of freshwater habitats, as at least 12 of the 31 insect orders have representatives occupying these environments during at least one life history stage [13]. Transitions between aquatic and terrestrial habitats are generally rarer than other habitat changes (e.g., between epigeal and arboreal) because of the substantial physical differences between them [14]. In addition to differences in the physical requirements of living in water versus on land (e.g., differences in oxygen concentration), one presumed barrier is that the suite of available food items are typically distinct, as there are major differences between aquatic and terrestrial food webs [15,16]. Therefore, in order to transition between these habitats, a lineage typically must adapt to new physical conditions while concomitantly modifying its feeding behaviors.

The family Sciomyzidae, or “snail-killing flies” (Diptera: Acalyptratae: Sciomyzoidea), is an ideal taxon with which to study the evolution of feeding behaviors and associated habitat transitions. Their life histories are well-studied, as 240 of the 539 species have known larval feeding habits [17]. Sciomyzid larvae display a wide range of feeding behaviors, including predation, parasitism, or saprophagy of terrestrial, semi-aquatic and aquatic non-operculate snails, operculate aquatic snails, semi-terrestrial succineid snails, slugs, snail eggs, fingernail clams and freshwater oligochaete worms [17,18]. This represents, by far, the most extensive radiation of primarily malacophagous (= mollusk-feeding) species when compared to all other dipteran lineages [17,18]. A total of 109 species from six other dipteran families attack mollusks [19], whereas ~99% of the 240 sciomyzid species with known life cycles attack mollusks [17,18,20]. Sciomyzids have three larval stages and most species exhibiting parasitoid behavior have very specific host requirements in the 1^st^ and 2^nd^ larval stage but become more generalized predators in the 3^rd^ stage. These species have been referred to as parasitoids or parasitoids/predators in sciomyzid literature, so, for simplicity, we refer to these species as parasitoids herein. There have been two different approaches to organizing sciomyzid species into behavioral/ecological groups: (*i*) based on commonalities in larval microhabitat, mode of feeding and prey type ([21]: 8 groups; [22]: 10 groups; [20]: 9 groups; [17,18]: 15 groups), and (*ii*) based on an ordination analysis of 36 egg and larval morphological characters, larval behaviors, and habitat that identified nine "Eco-Groups," each possessing a unique combination of states from these 36 characters [23].

The Sciomyzidae includes three subfamilies: the Huttonininae with two genera [24], the Salticellinae with one genus (*Salticella*) and the Sciomyzinae with the remaining 58 genera. The Sciomyzinae is comprised of two tribes, the Sciomyzini with 12 genera and the Tetanocerini with the remaining 46 genera [17]. All of the Sciomyzini and Salticellinae have terrestrial larvae, whereas 14 tetanocerine genera have at least one species with aquatic larvae [17]. The larvae of the Huttonininae remain unknown [17]. Recent phylogenetic analyses of morphological data suggest that the Sciomyzinae and its two tribes are monophyletic [23,25]. The family Phaeomyiidae, with five described Palaearctic species in two genera (*Akebono* and *Pelidnoptera*), was at one time considered to be a subfamily of the Sciomyzidae, but was subsequently elevated to family by Griffiths [26], who proposed its sister status to Sciomyzidae.

The evolution of feeding behaviors in Sciomyzidae has been discussed in numerous papers (e.g., [17,18,20-22]). Because larval feeding on decaying animal matter occurs in other dipteran lineages, including families in the Sciomyzoidea (e.g., Dryomyzidae; [17]), it has been suggested that the ancestral sciomyzid was probably similar to the extant *Atrichomelina pubera* (Sciomyzini), a generalist that feeds on dead, dying or living aquatic and semi-aquatic, non-operculate snails on damp terrestrial substrates [27,28]. Steyskal’s [29] classification of the Sciomyzidae lead to sciomyzine larvae being characterized as terrestrial (including those inhabiting moist surfaces) saprophages/predators/parasitoids, while tetanocerine larvae are typically characterized as aquatic predators. Knutson & Vala [18] mapped their feeding groups onto the morphological phylogeny presented in Marinoni & Mathis [25] to infer the ancestral feeding behavior for the family and to discuss the evolution of such behaviors based on the position of each genus in the phylogeny. They concluded that while Steyskal's [29] generalizations have exceptions, the distribution of feeding behaviors known today support these general characterizations. They further concluded that the terrestrial habits of many of the species in the Tetanocerini represent a derived condition within the tribe. Unfortunately, the utility of Knutson and Vala's [18] study was somewhat limited due to the incomplete resolution of intergeneric relationships and the absence of replicate intrageneric taxon sampling within the Marinoni & Mathis [25] phylogeny. A more recent study on intergeneric sciomyzid relationships [23], which included more morphological characters than did Marinoni & Mathis [25], was similarly limited, as the taxon sampling was nearly identical and the relationships among many of the genera not well-supported. Therefore, a well-resolved species-level phylogeny focusing on a lineage that exhibits a variety of feeding behaviors and occupies multiple habitats would enable a better understanding of the evolutionary processes involved in transitions among habitat, mode of feeding and host/prey selection.

Within the Tetanocerini, the genus *Tetanocera* is of particular interest because it is one of the most diverse sciomyzid genera with respect to feeding behaviors. Twenty-eight of its 39 species have known life cycles (see Table 1 for a partial list) and its species occupy five of the 15 feeding groups of Knutson & Vala [17,18]: (*i*) general predators of non-operculate aquatic snails in the water (14 species), (*ii*) general predators of non-operculate aquatic snails occurring on damp shorelines (3 species), (*iii*) general predators of terrestrial snails (2 species), (*iv*) parasitoids of slugs (4 species) or (*v*) parasitoids of succineid (semi-terrestrial) snails (5 species). The life cycles of 11 species remain unknown. Members of the largest feeding group within *Tetanocera* (*i* above) spend their larval stages just under the surface of the water, whereas the remaining groups have terrestrial larvae. Only one other sciomyzid genus occupies five feeding groups (*Sepedon*), whereas most only occupy one or two [17].

**Table 1:** **Species analyzed in this study, the feeding behavioral group**[17]**to which each taxon belongs, and GenBank numbers for the sequences used in this study**

**Family**	**Genus**		**Feeding Group**	**Specimen**	**GenBank Accession Numbers**				
** Tribe**	**Species**	**Feeding Group**	**Citation**	**Number**	**COI**	**COII**	**16S**	**28S**	**Ef-1α**
Drosophilidae	*Drosophila melanogaster* Meigen 1830	Yeast, mold	[80]		AJ400907	AJ400907	AJ400907	M21017	NM_170570
Phaeomyiidae	*Pelidnoptera nigripennis* (Fabricius 1794)	Millipede parasitoid	[81]	F272	JN860439			JN837497	JN816249
Sciomyzidae									
Sciomyzini	* Atrichomelina*								
	*Atrichomelina pubera* (Loew 1862)	Facultative predator/saprophage of snails and clams on damp shorelines	[28]	F160	JN860438	JN837567	JN816281	JN837498	
				F161	AY875151	AY875182	AY875089	AY875120	JN816247
	* Sciomyza*								
	*Sciomyza simplex* Fallén 1820	Predator of shoreline-stranded aquatics	[82]	F175	AY875152	AY875183	AY875090	AY875121	JN816248
Tetanocerini	*Anticheta*								
	*Anticheta melanosoma* Melander 1920	Predator of exposed snail eggs	[83]	F254	JN860440	JN837568	JN816327	JN837499	JN816250
	* Dichetophora*								
	*Dichetophora finlandica* Verbèke 1964	Unknown		F248	JN860441	JN837569	JN816328	JN837500	JN816251
	* Dictya*								
	*Dictya borealis* Curran 1932	Predator of aquatic snails in the water	[84]	F257	JN860442	JN837570	JN816329	JN837501	JN816252
	*Dictya expansa* Steyskal 1938	Predator of aquatic snails in the water	[84]	F263	JN860443	JN837571	JN816330	JN837502	
	*Dictya floridensis* Steyskal 1954	Predator of aquatic snails in the water	[85]	F258	JN860444	JN837572	JN816331	JN837503	
	*Dictya gaigei* Steyskal 1938	Predator of aquatic snails in the water	[84]	F267	JN860445	JN837573	JN816336	JN837504	
	*Dictya pictipes* (Loew 1859)	Predator of aquatic snails in the water	[84]	F261	JN860446	JN837574	JN816332	JN837505	JN816253
	*Dictya steyskali* Valley 1977	Predator of aquatic snails in the water	[84]	F270	JN860447	JN837575	JN816333	JN837506	
				F271	JN860448	JN837576	JN816334	JN837507	JN816254
	*Dictya stricta* Steyskal 1938	Predator of aquatic snails in the water	[84]	F260	JN860449	JN837577		JN837508	
	*Dictya texensis* Curran 1932	Predator of aquatic snails in the water	[84]	F268	JN860450	JN837578	JN816335	JN837509	
	*Dictyacium*								
	*Dictyacium firmum* Steyskal 1956	Unknown		F187	JN860451	JN837579	JN816337	JN837510	
				F188	JN860452	JN837580	JN816338	JN837511	
	*Elgiva*								
	*Elgiva connexa* Steyskal 1954	Predator of aquatic snails in the water	[86]	F150	AY875153	AY875184	AY875091	AY875122	
				F151	JN860453	JN837581	JN816282	JN837512	
				F152	JN860454	JN837582	JN816283	JN837513	JN816255
	*Elgiva solicita* (Harris 1780)	Predator of aquatic snails in the water	[86]	F5	AY875154	AY875185	AY875092	AY875123	
				F6	JN860455	JN837583	JN816284	JN837514	
	*Ethiolimnia*								
	*Ethiolimnia geniculata* (Loew 1862)	Unknown		F255	JN860456	JN837584	JN816339	JN837515	JN816256
	*Euthycera*								
	*Euthycera arcuata* (Loew 1859)	Parasitoid of slugs	[87]	F222	JN860457	JN837585	JN816340		JN816257
				F223	JN860458	JN837586	JN816341	JN837516	
				F224	JN860459	JN837587	JN816342	JN837517	
	*Hedria*								
	*Hedria mixta* Steyskal 1954	Predator of submerged aquatic snails	[88]	F168	JN860460	JN837588	JN816285	JN837518	
				F169	AY875155	AY875186	AY875093	AY875124	
	*Hoplodictya*								
	*Hoplodictya acuticornis* (Wulp 1897)	Parasitoid of succineid snails	LV Knutson (pers. comm.)	F277	JN860461	JN837589	JN816343	JN837519	JN816258
				F278	JN860462	JN837590	JN816344	JN837520	
	*Hydromya*								
	*Hydromya dorsalis* (Fabricius 1775)	Predator of shoreline-stranded aquatics	[89]	F249	JN860463	JN837591	JN816345	JN837521	JN816259
	*Ilione*		[90]						
	*Ilione albiseta* (Scopoli 1763)	Predator of submerged aquatic snails	[91,92]	F122	JN860464	JN837592	JN816286		
	*Limnia*								
	*Limnia boscii* Robineau-Desvoidy 1830	Parasitoid of succineid snails	LV Knutson (pers. comm.)	F120	AY875156	AY875187	AY875094	AY875125	JN816260
				F121	JN860465	JN837593	JN816287	JN837522	
	*Limnia ottawensis* Melander 1920	Unknown		F154	AY875157	AY875188	AY875095	AY875126	JN816261
	*Limnia sandovalensis* Fisher & Orth 1978	Unknown		F155	AY875158	AY875189	AY875096	AY875127	
				F156	JN860466	JN837594	JN816288	JN837523	
	*Pherbecta*								
	*Pherbecta limenitis* Steyskal 1956	Unknown		F237	JN860467	JN837595	JN816346	JN837524	JN816262
	*Pherbina*								
	*Pherbina coryleti* (Scopoli 1763)	Predator of shoreline-stranded aquatics	[37]	F250	JN860468	JN837596	JN816347		
	*Poecilographa*								
	*Poecilographa decora* (Loew 1864)	Unknown		F212	JN860469	JN837597	JN816348	JN837525	JN816263
				F230	JN860470	JN837598	JN816349	JN837526	
	*Psacadina*								
	*Psacadina zernyi* (Mayer 1953)	Predator of shoreline-stranded aquatics	[37]	F251	JN860471		JN816350	JN837527	JN816264
	*Renocera*								
	*Renocera amanda* (Cresson 1920)	Parasitoid of fingernail clams below the water's surface	[93]	F88	AY875159	AY875190	AY875097	AY875128	
	*Renocera johnsoni* (Cresson 1920)	Predator of aquatic snails in the water	BA Foote (unpublished)	F90	AY875160	AY875191	AY875098	AY875129	
				F92	JN860472	JN837599	JN816289	JN837528	JN816265
	*Renocera pallida* (Fallen 1820)	Parasitoid of fingernail clams above the water's surface	[94]	F193	JN860473	JN837600	JN816351	JN837529	
				F194	JN860474	JN837601	JN816352	JN837530	JN816266
	*Sepedon*								
	*Sepedon armipes* Loew 1859	Predator of aquatic snails in the water	[95]	F28	AY875161	AY875192	AY875099	AY875130	
	*Sepedon fuscipennis* Loew 1859	Unknown		F116	JN860475	JN837602	JN816360		
				F117	AY875162	AY875193	AY875100	AY875131	
	*Sepedon praemiosa* Giglio-Tos 1893	Predator of aquatic snails in the water	[95]	F118	AY875163	AY875194	AY875101	AY875132	JN816267
	*Tetanocera*								
	*Tetanocera amurensis* Hendel 1809	Unknown		F198	JN860478	JN837605	JN816290	JN837533	
				F199	JN860479	JN837606	JN816291	JN837534	
				F200	JN860480	JN837607	JN816292		
	*Tetanocera annae* Steyskal 1938	Predator of aquatic snails in the water	[96]	F201	JN860481	JN837608	JN816293		JN816270
				F202	JN860482	JN837609	JN816294		
				F229	JN860483	JN837610	JN816319	JN837535	
	*Tetanocera arnaudi* Orth & Fisher 1982	Unknown		F23	JN860484	JN837611	JN816295	JN837536	
				F24	JN860485	JN837612	JN816296	JN837537	
	*Tetanocera arrogans* Meigen 1830	Parasitoid of succineid snails	[39]	F93	AY875165	AY875196	AY875103	AY875134	JN816271
	*Tetanocera bergi* Steyskal 1954	Predator of aquatic snails in the water	[73]	F159	JN860486		JN816297	JN837538	JN816272
	*Tetanocera clara* Loew 1862	Parasitoid of slugs	[97,98]	F57	AY875167	AY875198	AY875105	AY875136	JN816273
	*Tetanocera elata* (Fabricius 1781)	Parasitoid of slugs	[99]	F245	JN860487	JN837613	JN816298	JN837539	
				F247	JN860488	JN837614	JN816299	JN837540	
	*Tetanocera ferruginea* Fallén 1820	Predator of aquatic snails in the water	[96]	F34	AY875168	AY875199	AY875106	AY875137	
				F158	AY875166	AY875197	AY875104	AY875135	
	*Tetanocera freyi* Stackelberg 1963	Unknown		F203	JN860489	JN837615	JN816300		JN816274
	*Tetanocera fuscinervis* (Zetterstedt 1838)	Predator of shoreline-stranded aquatics	[100]	F53	AY875169	AY875200	AY875107	AY875138	
				F54	JN860490	JN837616	JN816302	JN837541	
				F153	JN860491	JN837617	JN816301	JN837542	
	*Tetanocera hyalipennis* Roser 1840	Predator of shoreline-stranded aquatics	[39]	F127	JN860492	JN837618	JN816303		
				F191	JN860493	JN837619	JN816304	JN837543	
				F192	JN860494	JN837620	JN816305	JN837544	
	*Tetanocera kerteszi* Hendel 1901	Predator of terrestrial snails	LV Knutson (pers. comm.)	F46	AY875170	AY875201	AY875108	AY875139	
				F47	JN860495	JN837621	JN816306	JN837545	
	*Tetanocera latifibula* Frey 1924	Predator of aquatic snails in the water	[96]	F144	JN860496	JN837622	JN816357	JN837546	
				F146	JN860497	JN837623	JN816358	JN837547	
				F147	AY875171	AY875202	AY875109	AY875140	
				F149	JN860498	JN837624	JN816359	JN837548	
	*Tetanocera loewi* Steyskal 1959	Predator of aquatic snails in the water	[96]	F189	JN860499	JN837625	JN816307	JN837549	
				F226	JN860500	JN837626	JN816308	JN837550	
	*Tetanocera melanostigma* Steyskal 1959	Parasitoid of succineid snails	[101]	F2	AY875172	AY875203	AY875110	AY875141	
	*Tetanocera mesopora* Steyskal 1959	Predator of aquatic snails in the water	[96]	F40	AY875173	AY875204	AY875111	AY875142	
	*Tetanocera montana* Day 1881	Predator of aquatic snails in the water	[96]	F142	AY875174	AY875205	AY875112	AY875143	
				F143	JN860501	JN837627	JN816309	JN837551	
				F170	JN860502	JN837628	JN816310	JN837552	
				F171	JN860503	JN837629	JN816311	JN837553	JN816275
	*Tetanocera obtusifibula* Melander 1920	Predator of aquatic snails in the water	[96]	F275	JN860504	JN837630	JN816353	JN837554	
				F276	JN860505	JN837631	JN816354	JN837555	
	*Tetanocera oxia* Steyskal 1959	Parasitoid of succineid snails	[101]	F204	JN860506	JN837632	JN816312		
	*Tetanocera phyllophora* Melander 1920	Predator of terrestrial snails	[98,102]	F39	AY875175	AY875206	AY875113	AY875144	
	*Tetanocera plebeja* Loew 1862	Parasitoid of slugs	[97,98]	F1	JN860507	JN837633	JN816314	JN837556	
				F13	AY875176	AY875207	AY875114	AY875145	
				F205	JN860508	JN837634	JN816313		JN816276
	*Tetanocera plumosa* Loew 1847	Predator of aquatic snails in the water or on damp shorelines	[73]	F11	AY875177	AY875208	AY875115	AY875146	
				F43	JN860509	JN837635		JN837557	
	*Tetanocera robusta* Loew 1847	Predator of aquatic snails in the water	[96]	F10	AY875178	AY875209	AY875116	AY875147	
				F16	JN860510	JN837636	JN816317	JN837558	
				F134	JN860511	JN837637	JN816315	JN837559	JN816277
				F137	JN860512	JN837638	JN816316	JN837560	
	*Tetanocera rotundicornis* Loew 1861	Parasitoid of succineid snails	[101]	F206	JN860513	JN837639	JN816318	JN837561	
	*Tetanocera silvatica* Meigen 1830	Predator of shoreline-stranded aquatics	[100]	F35	JN860515		JN816321	JN837562	JN816279
				F172	AY875179	AY875210	AY875117	AY875148	JN816278
				F173	JN860514	JN837640	JN816320	JN837563	
	*Tetanocera soror* Melander 1920	Predator of aquatic snails in the water	[103]	F209	JN860516	JN837641	JN816322		
				F210	JN860517	JN837642	JN816323		
	*Tetanocera valida* Loew 1862	Parasitoid of slugs	[97,98]	F84	AY875180	AY875211	AY875118	AY875149	JN816280
	*Tetanocera vicina* Macquart 1843	Predator of aquatic snails in the water	[96]	F94	AY875181	AY875212	AY875119	AY875150	
				F95	JN860518	JN837643	JN816324	JN837564	
				F98	JN860519	JN837644	JN816325	JN837565	
				F99	JN860520	JN837645	JN816326	JN837566	
	*Trypetoptera*								
	*Trypetoptera canadensis* (Macquart 1843)	Predator of terrestrial snails	BA Foote (unpublished)	F164	AY875164	AY875195	AY875102	AY875133	JN816268
* Trypetoptera punctulata* (Scopoli 1763)	Predator of terrestrial snails		[104]	F217	JN860476	JN837603	JN816355	JN837531	JN816269
				F218	JN860477	JN837604	JN816356	JN837532	
Sequence coverage				out of 114 OTUs		out of 65 species		out of 23 genera	
COI				114: 100%		65: 100%		23: 100%	
COII				110: 96.5%		62: 95.4%		21: 91.3%	
16S				111: 97.4%		63: 96.9%		22: 95.6%	
28S				101: 88.6%		60: 92.3%		21: 91.3%	
EF1α				34: 29.8%		33: 50.8%		19: 82.6%	

In a previous paper, a DNA sequence-based phylogeny of sciomyzids was used to examine the evolution of larval characters that appeared correlated with larval habitat [30]. Character states in four larval characters were found to be significantly correlated with aquatic to terrestrial transitions in *Tetanocera* where each larval character changed in the same way as multiple lineages made independent habitat transitions. In the present study, we build on these findings by examining feeding behavior evolution, as feeding behaviors are dependent on both larval morphological adaptations to different environments and specific requirements related to finding and subduing different prey species. Given the diversity of feeding behaviors within Sciomyzidae and *Tetanocera*, it is important to determine whether there were single or multiple origins of feeding behaviors. Such an analysis would simultaneously show whether there was convergent evolution of larval habitat and the relative frequencies of habitat transitions. Multiple evolutionary hypotheses regarding feeding behaviors and habitat transitions are presented in the literature (e.g., [17,18,20,21,23,27-29]) and all should be considered plausible until rigorously evaluated using modern phylogenetic comparative methods. Therefore, the present study has four specific objectives: (*i*) construct a robust estimate of phylogeny for *Tetanocera* and Tetanocerini based on multiple mitochondrial and nuclear genes, (*ii*) estimate the evolutionary transitions in larval feeding behaviors, habitats and host/prey that have occurred during the evolution of Tetanocerini and *Tetanocera*, (*iii*) test prior hypotheses regarding the monophyly of feeding and ecological groupings and (*iv*) identify the mechanisms underlying habitat and feeding behavior evolution in *Tetanocera*.

## Results

### Phylogenetic analyses

**Figure 1:**
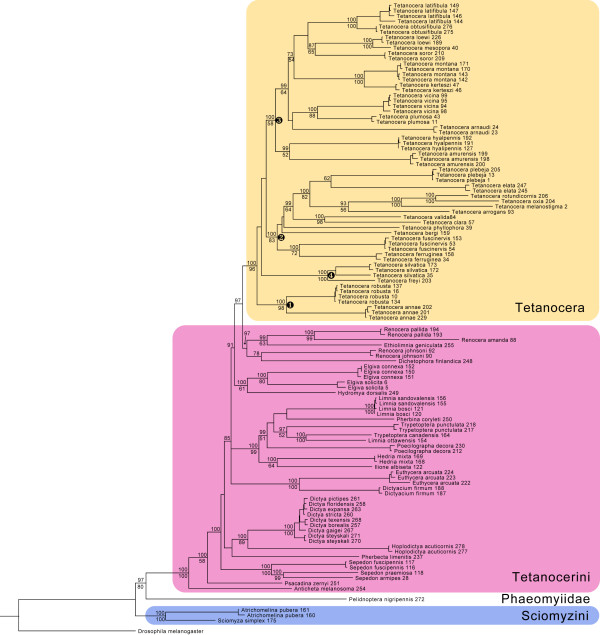
** Majority rule consensus of 20,000 post burn-in trees from a 160 million generation Bayesian analysis of COI, COI and 16S mtDNA and 28S nuclear DNA from 64 sciomyzid and one phaeomyiid species under a partitioned substitution model.** Bayesian posterior probabilities (x100) appear above the nodes and maximum likelihood bootstrap values (200 bootstrap replicates) appear below the nodes. Nodal support values for individuals of the same species were generally high, but were left off due to spatial constraints (as were those for species of *Dictya*), but appear in the supplemental figures. *Drosophila melanogaster* sequences were used to root the analysis. Numbers after species names are specimen numbers (Table 1).

We used Bayesian inference (BI) and maximum likelihood (ML) to analyze a concatenated 5-gene data set. The BI MAP tree with BI posterior probabilities (x100) and ML bootstrap nodal support values is shown in Figure 1. The BI MAP and best ML tree (Additional file 1: Figure S1) were largely congruent (also see Additional file 1: Figure S2, Additional file 1: Figure S3 for BI consensus and ML bootstrap trees, respectively). Both recovered a monophyletic Tetanocerini (BI PP = 1.0; ML bootstrap = 100), a monophyletic Sciomyzini (PP = 1.0; BS = 100), and placed *Pelidnoptera*, now in Phaeomyiidae but once considered a subfamily of Sciomyzidae (e.g., [31]) as the sister lineage to the Tetanocerini, suggesting its potential status as a tribe within the Sciomyzinae (PP = 0.97; BS = 80; Figure 1). All genera with multiple species are monophyletic except for (1) *Limnia*, which is rendered polyphyletic by *Trypetoptera* and *Pherbina* in the BI MAP tree and by *Trypetoptera* in the best ML tree, (2) *Trypetoptera*, rendered polyphyletic by *Limnia ottawensis* in both trees and (3) *Renocera*, rendered polyphyletic by *Ethiolimnia* and *Dichetophora* in both trees. The polyphyly of these genera are each supported by at least one node with high BI PP and ML BS values (Figure 1).

**Figure 2:**
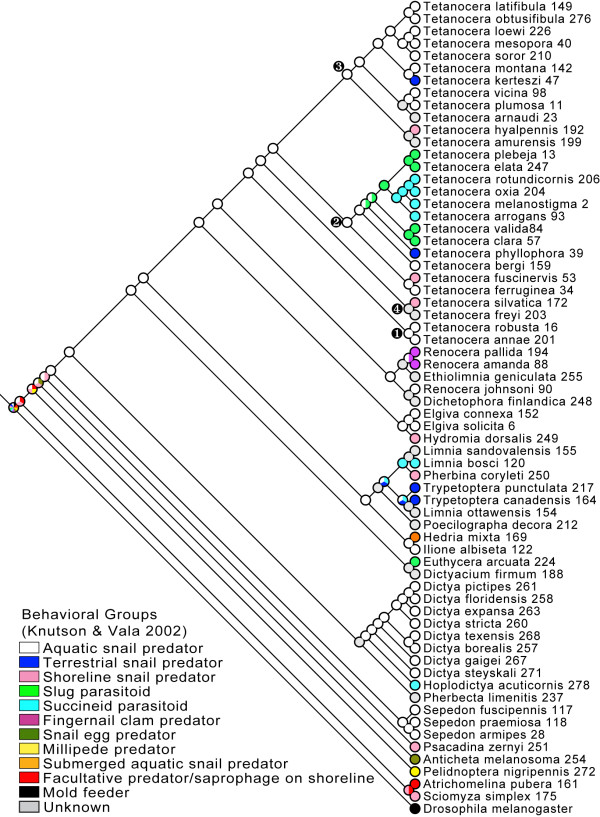
** Maximum likelihood optimization of Knutson and Vala's**[17]**larval feeding groups on the topology from Figure**1**(pruned to include only one terminal per species) analyzed with Mesquite using the MK1 model of character evolution.** Only character states that are statistically significantly better than the others (ancestral character state estimates with a log likelihood two or more units higher than all others) are shown in the pie charts at the nodes. A solid (one color) node indicates that state is significantly better than all other possible states. Grey indicates unknown character states. Numbers after species names are specimen numbers (Table 1).

**Figure 3:**
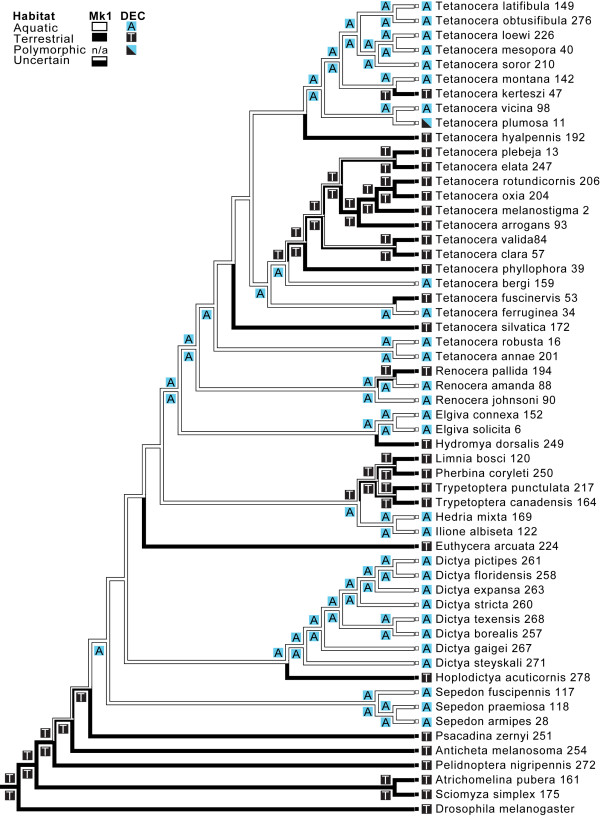
** Maximum likelihood optimization of larval habitat on the topology from Figure**1**(pruned to include only one terminal per species) analyzed with Mesquite using the AsymmMk model of character evolution.** Only character states that are statistically significantly better than the others are shown along the branches. A solid (one color) node indicates that state is significantly better than all other possible states. Numbers after species names are specimen numbers (Table 1). Lagrange-estimated ancestral charater states are denoted by blue (aquatic) and black (terrestrial) boxes. Only those nodes with a single state estimated to be significantly better than all other states are plotted. The full Lagrange output is shown in Additional file 1: Figure S5.

Both BI and ML recovered a monophyletic *Tetanocera* (PP = 1.0; BS = 96; Figure 1). Within *Tetanocera*, both trees have *T. robusta* + *T. annae* (Figures 1,2,3: clade ➀) as sister to the remaining species. Both analyses recovered *Tetanocera* clade ➁ (Figures 1,2,3) with identical relationships. This clade includes all five behavioral groups known for the genus (Table 1; Figure 2). The other major *Tetanocera* clade common to both trees (Figures 1,2,3: clade ➂) contains eight aquatic predators, one shoreline predator, one terrestrial predator and two species with unknown life cycles, with relatively minor differences in species relationships between the BI and ML trees. Finally, both analyses recovered *T. silvatica* + *T. freyi* (Figures 1,2,3: clade ➃) as sister species: in the BI MAP tree, clade ➃ is sister to clade ➁ + clade ➂, however in the best ML tree, clade ➃ is sister to clade ➂ (Additional file 1: Figure S1). The BI MAP and best ML trees were not significantly different from one another as judged by ML methods via GARLI and CONSEL (Table 2).

**Table 2:** Results of the likelihood-based approximately unbiased (AU), Shimodiara-Hasegawa (SH), weighted Kishino-Hasegawa (WKH), and weighted Shimodiara-Hasegawa (WSH) tests calculated using CONSEL

			**Test**			
**Constraint**	**-ln L**	**Difference**	**AU**	**SH**	**WKH**	**WSH**
*Tetanocera* feeding group analysis (59-taxon data set)						
Unconstrained	−38932.126	(Best)				
Aquatic snail predators1*	−39212.682	280.556	**p = 4e-06**	**p < 1e-100**	**p < 1e-100**	**p < 1e-100**
Aquatic snail predators2*	−39252.447	320.321	**p = 2e-41**	**p < 1e-100**	**p < 1e-100**	**p < 1e-100**
Shoreline snail predators1	−38971.538	39.412	**p = 0.001**	**p = 0.002**	**p = 0.002**	**p = 0.002**
Shoreline snail predators2*	−39004.527	72.401	**p = 1e-07**	**p = 0.005**	**p < 1e-100**	**p = 4e-05**
Slug parasitoids	−38941.015	8.889	**p = 0.038**	p = 0.631	p = 0.062	p = 0.193
Terrestrial snail predators*	−39051.229	119.103	**p = 1e-08**	**p < 1e-100**	**p < 1e-100**	**p < 1e-100**
Renocerinae monophyly analysis (entire 115-taxon data set)						
Unconstrained	−73022.049	(Best)				
Renocerinae	−73056.937	34.89	p = 0.055	p = 0.059	p = 0.059	p = 0.059
Comparison of Bayes MAP (Figure 1) and best ML (Additional file 1: Figure S1) trees (entire data set)						
ML tree	−72999.441	(Best)				
Bayes MAP tree	−73005.315	5.875	p = 0.377	p = 0.388	p = 0.388	p = 0.388

Knutson and Vala [17] feeding group constraints were done with an abbreviated data set containing 59 terminal taxa (all *Tetanocera* plus 4 outgroups). Trees compared were the best topology from unconstrained analysis versus an analysis where the feeding groups (see Table 1) were constrained to be monophyletic. *Tetanocera plumosa*, which can either live in the water or on the shoreline was coded both ways (Aquatic2 & Shoreline2 = *T. plumosa* considered a shoreline snail predator). The monophyly of *Anticheta + Renocera,* proposed as subfamily Renocerinae by Verbeke [74], was tested by constraining them to be outside of the Tetanocerini and Sciomyzini. The Bayesian MAP tree and ML tree were tested to see if they were significantly different from one another. P-values in bold are significant. Constraints with an asterisk (*) were constrained trees that were significantly worse than the unconstrained tree in all statistical tests.

### Behavioral group optimizations

We optimized Knutson and Vala's [17] larval behavioral groups on the BI MAP tree using ML methods in Mesquite (Figure 2; see Additional file 1: Figure S4 for optimization on the best ML tree). From these optimizations, we infer that (*i*) the evolution of aquatic larvae occurred relatively early in tetanocerine phylogenesis, (*ii*) from this aquatic ancestor, at least 10 lineages made independent, evolutionary reversals to terrestrial existence and, during the process, made at least 15 feeding behavioral transitions, (*iii*) the ancestor of *Tetanocera* was a general predator of non-operculate snails just below the water surface and (*iv*) a minimum of five *Tetanocera* lineages made independent, evolutionary reversals to terrestrial existence during which at least seven transitions in feeding behaviors occurred. All of these transitions were judged significant by ML criteria. The optimization of larval habitat (Figure 3) demonstrates an identical aquatic to terrestrial transition pattern (as compared to Figure 2) within the Tetanocerini subsequent to the divergence of *Sepedon*. Removal of species with unknown life cycles had no significant effects on either optimization.

**Figure 4:**
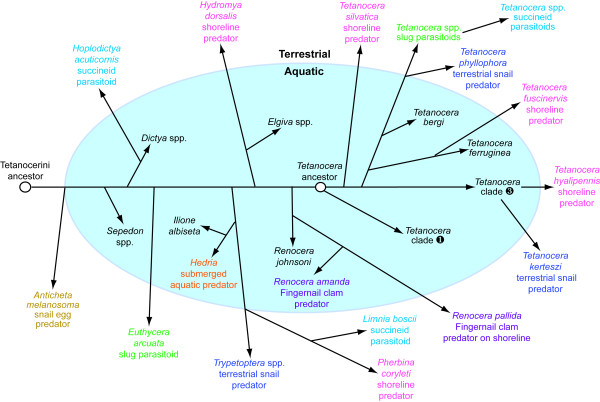
**Diagram showing the evolution of feeding behaviors and habitat changes in the Tetanocerini based on the topology and optimization of Knutson and Vala's**[17]**behavioral groups in Figure**2**(unknowns removed).** Aquatic lineages in black type are all general predators of aquatic snails. Every line that crosses from blue to white background represents an aquatic-to-terrestrial transition. Branches that split at the aquatic-terrestrial interface indicate uncertainty of ancestral habitat

We also estimated the evolution of habitat changes using the dispersal-extinction-cladogenesis (DEC) model implemented in the program Lagrange [32]. We found the optimal ratio of aquatic-to-terrestrial vs. terrestrial-to-aquatic transitions was between 11:1 and 13:1 which was significantly better than the null model with no bias in habitat transition rates (i.e., the global ML estimate was more than two log-likelihood units higher; Additional file 1**:** Table S1) and congruent with the Mesquite optimization. Therefore, the DEC model-estimated ancestral states plotted on Figure 3 are those with Lagrange set to a 12:1 ratio of aquatic-to-terrestrial vs. terrestrial-to-aquatic transitions. This procedure significantly estimated a terrestrial habitat for the sciomyzid ancestral lineage, a single terrestrial-to-aquatic transition and five unambiguous aquatic-to-terrestrial transitions. Furthermore, both the DEC and Mesquite optimizations are congruent and complementary such that only one node, near the base of the tree, does not have a significant habitat estimation (Figure 3). The tetanocerine feeding behavior and habitat transitions based on the Mesquite optimizations in Figures 2 and 3 are better visualized in Figure 4.

### Constraint analyses

To test hypotheses of multiple independent feeding behavior and habitat transitions, ML analyses were performed in which the monophyly of each polyphyletic *Tetanocera* behavioral group was constrained (Table 2). In the analyses where (1) aquatic snail predators (two variations), (2) stranded shoreline snail predators (two variations) and (3) terrestrial snail predators were constrained, all topology tests rejected their monophyly as the constrained trees yielded significantly lower log-likelihoods than the unconstrained trees (Table 2). These results support the hypothesis that there were multiple independent transitions from aquatic snail predation in the water to (1) aquatic snail predation on the shoreline and (2) terrestrial snail predation. Constraining the monophyly of slug parasitoid *Tetanocera* species did not result in a significantly different topology from the unconstrained tree. Finally, constraining *Anticheta* + *Renocera* to be monophyletic and further constrained to be a separate lineage outside of the currently recognized tribes produced a tree that was not significantly worse than the unconstrained tree (Table 2).

## Discussion

### Evolutionary transitions in sciomyzid larvae

Based on life history and larval morphological studies, Knutson & Vala [18] concluded that “terrestrial behavior and morphology in the Tetanocerini are apomorphic features of that tribe.” The present study and others support this conclusion. Wiegmann *et al*. [33] performed phylogenetic analyses of a comprehensive dipteran data set that yielded a monophyletic Sciomyzoidea that included eight families currently classified in the Sciomyzoidea, the Huttoninidae (elevated to family) and Conopidae, a family not previously included in the Sciomyzoidea (also see [34]). The Sciomyzidae occupies a relatively derived position within the Sciomyzoidea in the Wiegmann *et al*. [33] phylogeny. The only sciomyzoid taxon known to contain aquatic larvae is the Tetanocerini. Therefore, higher-level studies of Sciomyzoidea [33] support a terrestrial ancestor for Sciomyzidae and their closest relatives. The derived position of Tetanocerini within Sciomyzidae (Figures 1,2) suggests the freshwater aquatic habit is a unique derived feature of this clade, and terrestrial behavior and morphology in the Tetanocerini are largely derived from aquatic ancestry. Our habitat optimizations (Figure 3) strongly support a terrestrial ancestor for Sciomyzidae and both Mesquite optimizations (Figures 2,3) strongly support an aquatic ancestor for Tetanocerini minus *Anticheta* and *Psacadina* (i.e., the third basal-most Tetanocerini node). These analyses clearly demonstrate a (*i*) sciomyzid ancestor with terrestrial larva, (*ii*) derived aquatic habitat for the ancestor of most Tetanocerini and (*iii*) secondarily derived terrestrial habitat for multiple tetanocerine lineages.

The genus *Tetanocera* is one of the most remarkable sciomyzid genera with respect to feeding behaviors, microhabitat and host/prey preference as its species are members of five of Knutson and Vala's [17] behavioral groups. The monophyly of *Tetanocera* is well-supported (BI PP = 1.0; ML bootstrap = 96; Figure 1, Additional file 1: Figure S1), and the ancestor to *Tetanocera* was strongly suported as being a predator of aquatic snails (Figure 2). From this ancestral condition, there were three independent transitions to shoreline predation on aquatic snails, two independent transitions to terrestrial snail predation, and one transition to slug parasitoidism with one lineage subsequently transitioning to succineid snail parasitoidism (Figures 2,4). Furthermore, the monophyly of three of these behavioral groups has been rejected (Table 2). In a more general sense, this implies that *Tetanocera* lineages made between three (Lagrange) and five (Mesquite) independent transitions to terrestrial habitats (Figure 3). These transitions were estimated to be statistically significant by ML (Figures 2,3, Additional file 1: Figure S4, Additional file 1: Figure S5). In their modification of Steyskal’s [35] morphology-based, *Tetanocera* species groups, Boyes *et al.*[36] stated that “the derived, terrestrial modes of [feeding] behavior have clearly arisen several times in different species groups.” Our species-level phylogeny, ML optimizations and topology tests clearly support this conclusion.

### Sciomyzids violate trends in habitat transitions

In their study of evolutionary aquatic-terrestrial habitat transitions, Vermeij and Dudley [14] concluded that with the exception of tetrapod vertebrates, aquatic-to-terrestrial habitat transitions are rare as compared to the reverse. However, within Diptera, more than 20 lineages have made such transitions (inferred from Figure 1 of [33]). From the present study, it can be concluded that sciomyzid lineages have made an exceptional number of independent transitions between aquatic and terrestrial habitats. Specifically, they have made at least one transition from terrestrial to aquatic habitats, with Mesquite estimating at least 10 lineages subsequently experiencing evolutionary reversals to terrestrial habitats (Figures 2,3, Additional file 1: Figure S4). Additionally, the Lagrange analysis demonstrated a significant bias in the transition rate towards aquatic-to-terrestrial transitions with the overall log-likelihood maximizing near a ratio of 12:1 (Additional file 1: Table S2). Furthermore, some species in each of nine tetanocerine genera not included in our analyses (*DichetophoraDictyaEulimniaNeolimniaPerilimniaProtodictyaSepedonSepedonellaShannonia*) have terrestrial larvae [17], so it is quite likely that there have been additional aquatic-to-terrestrial transitions during the phylogenesis of the Tetanocerini. If we assume that *DichetophoraDictyaNeolimniaProtodictya* and *Sepedon* are monophyletic and arose after the early tetanocerine ancestor entered the water (obviously true for three of these genera in Figure 1, Additional file 1: Figure S1), then as many as five additional aquatic-to-terrestrial transitions have occurred, because each of these genera have both aquatic and terrestrial members [17,18]. Given that life cycle, habitat and host/prey information is available for only 240 of 540 sciomyzid species and 41 of the 61 genera [17], the actual number of independent aquatic to terrestrial habitat transitions could easily number in the 20s. Clearly, aquatic-to-terrestrial habitat transitions are strikingly common in the Sciomyzidae.

### Possible mechanisms for aquatic to terrestrial transitions

Vermeij and Dudley [14] also concluded that predation intensities are generally lower in freshwater habitats than they are on land, therefore offering less biotic resistance to transitions from terrestrial to freshwater habitats than the reverse. However, our estimated evolutionary transitions within the Tetanocerini show the opposite pattern, with a 10:1 ratio of aquatic-to-terrestrial vs. terrestrial-to-aquatic transitions (Figures 2,3,4; Additional file 1: Table S2). This raises the question of why sciomyzids are going against the trend observed by Vermeij and Dudley [14]. A portion of the answer likely lies in the larval morphological adaptations necessary for survival in each habitat. Chapman *et al.*[30] examined changes in four larval characters that were found to be significantly correlated with aquatic-to-terrestrial transitions in *Tetanocera*. They found that in each independent transition, the larvae of terrestrial lineages experienced reductions or losses in three characters associated with breathing while under water and lost pigmentation (also see [37]). This trend was observed across the Tetanocerini by Vala and Gasc [38], who found a series of reductions in the same breathing-related characters as lineages moved from aquatic to shoreline to drier terrestrial habitats. In order for a terrestrial Tetanocerini lineage to enter the water, it would have to gain those adaptations necessary to respire while mostly submerged. Therefore, the relative ease of losing aquatic adaptations versus the relative difficulty of gaining such adaptations *de novo* is likely one of the primary reasons that there is a much higher rate of aquatic to terrestrial habitat transitions than the reverse in sciomyzids. This significant reduction in aquatic-to-terrestrial adaptive morphological constraints indicates that tetanocerine phylogenesis likely tracked some ecological pressures (e.g., increased aquatic predation and/or increased terrestrial food availability) more accurately than did more constrained lineages.

In order for a lineage to make a successful transition to a new habitat, the members must be able to compete for and acquire resources in the new habitat [14]. Generally, it seems rather unlikely that a lineage could make multiple parallel transitions into a new habitat as the success of these transitions would typically depend upon a simultaneous adaptation to new physical conditions as well as the utilization of new food resources. However, in the case of the Tetanocerini, the intermediate nature of the damp, shoreline habitat likely played a significant role in facilitating parallel aquatic-to-terrestrial habitat transitions. Our analyses demonstrate that five of the 10 independent transitions to terrestrial habitats were to the shoreline habitat where the prey taxa are the same as their aquatic ancestors (*HydromyaRenoceraTetanocera*; Figures 2,4). Both aquatic snails and fingernail clams occur on damp shorelines where they are either periodically stranded by receding or fluctuating waters, or in the case of snails, temporarily foraging out of the water or migrating between aquatic habitats [17]. The availability of the same food resources on the shoreline as in the water likely facilitated stepwise transitions to terrestrial existence in multiple sciomyzid lineages. Like aquatic snails, aquatic sciomyzid larvae can move onto the damp shoreline in search of their prey. However, shoreline-adapted species have lost their adaptations to breathing while under the surface of the water, and will actively swim to shore if placed in water [39]. Once a lineage has adapted to living on damp shorelines (possessing only vestiges of the adaptations to breathing while under water), they may find it more difficult to go back into the water (having to re-express the aquatic-adapted traits) than to move to even drier habitats or switch prey type. Once accustomed to feeding on aquatic snails on terrestrial shorelines, tetanocerine lineages then are pre-adapted to preying on non-aquatic gastropods. Relative to the Tetanocerini, derived terrestrial food items include slugs, succineid (semi-terrestrial) snails and land snails. Given this evolutionary scenario, it is easy to imagine how the ancestors of dry-land terrestrial snail-feeding lineages like *TrypetopteraTetanocera phyllophora* and *T. kerteszi* used the shoreline as a stepping-stone environment facilitating a gradual movement to dry-land habitats where they became generalist predators of land snails.

A central question remains as to what selection pressures led multiple lineages of Tetanocerini to transition to terrestrial habitats with some lineages switching to prey other than aquatic pulmonate snails. Chapman *et al.*[30] speculated that it was a combination of (1) eliminating competition with other aquatic snail predators, (2) compensating for prolonged declines in aquatic snail populations, (3) escaping aquatic insect predators/parasitoids and (4) the reduction and/or loss of suitable aquatic habitats due to the general drying climatic trend that took place between 65 and 5 mya (beginning of Cenozoic era to the end of the Pliocene epoch) that drove these terrestrial transitions. Wiegmann *et al.*[33], using the penalized likelihood method in r8s [40], estimated that the Sciomyzidae originated ~30 mya (see Fig. S3 in [33]). The oldest known fossil Tetanocerini are preserved in Baltic amber (55–24 mya [41]), and the oldest known fossil *Tetanocera* (although this generic assignment is questionable [41]) is from the Oligocene epoch (34–24 mya [42,43]). These data place Tetanocerini lineages within the general drying period mentioned above. Therefore, (*i*) the relative ease of reducing or losing morphological characters (compared to gaining them *de novo*), (*ii*) the occurrence of the same prey on damp shorelines as occur in the water and (*iii*) the general drying trend all likely played key roles resulting in multiple tetanocerine lineages making independent aquatic-to-terrestrial transitions during their phylogenesis.

### General implications for evolutionary transitions

The results of this study may have implications for how changes between aquatic and terrestrial habitats have occurred in other animals. Any lineage that (1) occurs in aquatic and terrestrial habitats, (2) respires the same way in aquatic and moist shoreline habitats (e.g., cuticular respiration or open tracheal system) and (3) has the same type of food available in both habitats (e.g., pulmonate snails) could show a similar pattern of multiple independent habitat transitions coincident with changes in behavioral and morphological traits. Borda & Sidall [44] found multiple aquatic-to-terrestrial transitions in arynchobdellid leeches, and Rubinoff [45] found either multiple independent terrestrial-to-aquatic transitions or an evolutionary reversal to terrestrial habitats in one lineage of cosmopterigid moths in Hawaii. Both of these taxa fit the above criteria. Like Sciomyzidae, at least 34 other dipteran families have both aquatic and terrestrial lineages [33] and many of the larger such families have larvae that are, in general, restricted to air-breathing (e.g., Culicidae, Dixidae, Dolichopodidae, Stratiomyidae, Syrphidae, Tipulidae and Tabanidae [13]). Air-breathing insects have open tracheal systems and must establish contact between their spiracles and the atmosphere to respire and must therefore either remain at or come to the surface periodically. Of these families, the Tipulidae (crane flies), unlike many of the families traditionally classified in the suborder Nematocera (primitive flies with long, filamentous antennae) that probably share an aquatic ancestor, may have originated in damp terrestrial, tropical habitats [11]. Wiegmann *et al*.'s [33] plot of aquatic habitat on their comprehensive dipteran phylogeny indicated that most of the families of suborder Brachycera (derived flies with short antennae) with aquatic lineages were likely of terrestrial origin. Therefore, the findings presented herein should broadly interest anyone studying the evolution of aquatic and terrestrial habitat transitions and associated behavioral and morphological changes in Diptera, a group that includes over 152,000 currently named species [33]. Other lineages that fit the above criteria include oligochaete worms, pulmonate gastropods, decapods, isopods, amphipods, orbatid mites, true bugs in the infraorder Nepomorpha and beetles in the suborder Adephaga, superfamily Byrrhoidea and family Lampyridae. The results of the present study are suggestive that some lineages within these groups will also show multiple convergences on aquatic or terrestrial habits when examined with modern phylogenetic comparative methods.

## Conclusions

Phylogenetic analyses of sciomyzid DNA sequences provided strong support that the Sciomyzini, Tetanocerini and *Tetanocera* are monophyletic (Figure 1). We significantly estimated that (i) the ancestor of the Sciomyzidae was terrestrial (Figures 2, 3), (ii) there was a single terrestrial-to-aquatic transition early in the evolution of the Tetanocerini and, subsequently, (iii) there were at least 10 independent aquatic-to-terrestrial transitions and at least 15 transitions in feeding behaviors (Figures 2, 3, 4, Additional file 1: Figure S2). The 10:1 ratio of aquatic-to-terrestrial vs. terrestrial-to-aquatic transitions goes against the general trend observed in animals. We found that the ancestor to *Tetanocera* was aquatic and five *Tetanocera* lineages made independent aquatic-to-terrestrial transitions and seven independent transitions in feeding behaviors (Figures 2, 3, Additional file 1: Figure S2). Classifications of sciomyzids into ecological assemblages of species resulted in many non-monophyletic groupings (Figures 2, 3, 4, Additional file 1: Figure S2, Additional file 1: Figure S3) whose monophyly were rejected via phylogenetic constraint analyses (Table 2). Therefore, these findings strongly support our inferences of multiple independent transitions in feeding behaviors, habitats and prey/host usage. The damp shoreline habitat is likely a crucial transitional habitat where tetanocerine lineages that move out of the water to forage can find the same prey taxa as in the water. Once tetanocerine lineages are established on the shoreline, terrestrial molluscan taxa are available as potential food resources. From a morphological standpoint, transitioning from aquatic to terrestrial habitats is easier than the reverse, as adaptations to air-breathing just below the surface of the water are more difficult to gain than to lose. Furthermore, tetanocerine phylogenesis occurred as the Earth was going through a general drying period. These factors likely explain why so many tetanocerine lineages made secondary transitions to terrestrial environments. Finally, the results herein imply that any animal lineage that has aquatic and terrestrial members, respire the same way in both habitats and have the same type of food available in both habitats could show a similar pattern of multiple independent habitat transitions coincident with changes in behavioral and morphological traits.

## Materials and methods

### Taxon sampling

Phylogenetic analyses were performed on DNA sequences from five genes obtained from 60 *Tetanocera* specimens (representing 28 species) and 53 individuals representing 21 additional genera within the Sciomyzidae (19 from the Tetanocerini (34 species)), two from the Sciomyzini (2 species) and *Pelidnoptera* (Phaeomyiidae) which is not currently considered to be a member of the Sciomyzidae but is thought to be its sister taxon ([23,25,26] but see [33]). Therefore, our analyses include 72% of *Tetanocera* species, 42% of the genera of Tetanocerini, and 15% of the genera of Sciomyzini. *Drosophila melanogaster* (Drosophilidae) was used as the outgroup in all unconstrained phylogenetic analyses. Table 1 contains a complete listing of the taxa analyzed in this study including GenBank accession numbers and the percentage of OTUs, species and genera sequenced for each gene. For 18 of the 28 *Tetanocera* species, multiple individuals were available and sequenced for replicate sampling purposes. Of the 29 *Tetanocera* species with known life cycles, 25 are examined. Of the 41 sciomyzid genera that have behavioral information known for the larvae of at least one species, at least one representative of 17 genera is included. Ten of Knutson and Vala's [17] 15 feeding groups are represented.

### Laboratory protocols

Field collections of adult specimens were preserved immediately in 95% ethanol. In the laboratory, specimens were transferred to vials containing 100% hexamethyldisilazane (Polysciences, Inc., Warrington, Pennsylvania, USA) for at least 24 hours, after which the liquid was decanted and the specimens allowed to dry under a fume hood. Prior to preparation for total DNA isolation, the head, legs, wings and abdomen of each specimen were removed from the thorax. Total DNA was isolated from each thorax, and the remaining body parts (which contain the morphological characters necessary for species determination) are stored as vouchers in 95% ethanol at the University of Kentucky. Each specimen and associated DNA extraction was given a unique number. Species identification, collecting locality information and habitat notes were recorded in a database.

Total DNA was isolated from single individuals using Qiagen DNeasy Tissue Kits (QIAGEN Inc., Chatsworth, California, USA) following the manufacturer’s animal tissue protocol. We PCR-amplified fragments of the mitochondrial cytochrome *c* oxidase subunits I (*COI*) and II (*COII*) and *16S* rDNA genes, and the nuclear *28S* rDNA and elongation factor-1 alpha (*EF-1α*) genes using the primer pairs listed in Table 3. Each amplicon was purified in NuSieve® GTG® low melting temperature agarose (Lonza, Rockland, Maine, USA) and separated from the agarose with Wizard® PCR preps DNA purification system (Promega Corp., Madison, Wisconsin, USA). PCR reactions (total volume = 50 μL) consisted of 1X Qiagen PCR buffer, 0.2 mM of each dNTP, 0.5 mM of each primer, 1.25 U of Qiagen *Taq* and 1–5 μL of template DNA. Cycle sequencing protocols followed Folmer *et al.*[46]; both strands were cycle sequenced using either end-labeled primers (Perkin Elmer AmpliCycle Sequencing Kits; Li-COR sequencer) or labeled dideoxynucleotides (ABI Big-Dye Terminator mix v. 3.0; Applied Biosystems, Foster City, California, USA; ABI sequencer). The separation of cycle sequencing reaction products was done in 3.7% and 5.5% polyacrylamide gels in LI-COR 4200 L-2 and 4200S-2 automated DNA sequencers, respectively, or Applied Biosystems 3730XL or 3730 DNA Analyzers.

**Table 3:** Genes / primer information used in this study

**Gene**	**Primer pair**	**References**	**Analyzed fragment size**	**Notes**
Mitochondrial loci:				
16S	LR-N-13398 / LR-J-12887	[105]	426 bp	Primer sequences identical to those of “Locust”
COI	LCO1490	[46]	658 bp	Together, both COI primer pairs encompass nearly the entire gene
	HCO-700ME	[106]		
	C1-J-2183 / TL2-N-3014	[105]	813 bp	
COII	TL2-J-3034 / TK-N-3785	[105]	681 bp	Amplify all of COII
Nuclear loci:				
28S	D1F / D6R	[107]	1095 bp	
Ef-1α	ScioEF1a-F	Designed herein	876 bp	CAYMGDGATTTCATYAARAACATGA
	ScioEF1a-R			GCRATGTGAGCGGTGTGRCAATCC

Analyzed fragment size is the number of base pairs remaining after primer sequences and regions of ambiguous alignment were removed.

### Phylogenetic analyses

Bi-directional sequences were aligned using AlignIR (v. 2.0, LI-COR Biosciences, Inc., Lincoln, Nebraska, USA). Multiple sequence alignments of each gene region were produced with MAFFT [47]. The alignments of the *COI, COII* and *EF-1α* sequences contained no indels, however, indels that presented alignment ambiguities were found in the sciomyzid *16S* and *28S* sequences. The GUIDANCE server [48] was used to assess confidence scores for each column in the MAFFT alignments. Columns with confidence scores < 95% were removed prior to all phylogenetic analyses. The data sets analyzed herein (including program-specific commands) have been deposited on Dryad (http://dx.doi.org/10.5061/dryad.cb098).

Bayesian inference (BI) phylogenetic analyses were conducted on a concatenated (using MacClade v. 4.08 [49]) 4,549-character data set (*COI* = 1471 nt, *COII* = 681 nt, *16S* = 426 nt, *28S* = 1095 nt, *EF-1α* = 876 nt) with MrBayes (v. 3.1.2 [50,51]). The data set contained 114 terminal taxa for which we generated sequences (including 31 terminals from Chapman *et al*. [30]), plus one additional terminal (*D. melanogaster*) whose sequences were obtained from GenBank (Table 1). The data were partitioned by gene region and codon position when appropriate (11 total partitions: three gene regions × three codon positions for the *COICOII* and *EF-1α* partitions plus a single partition each for *28S* and *16S*) and jModeltest (v. 12.9.0 [52]) was used to determine the best-fit model for each partition (Additional file 1: Table S2). To allow each partition to have its own set of parameter estimates, *revmattratiostatefreqshape*, and *pinvar* were all unlinked during the analyses. To obtain the most accurate branch length estimates possible, the option *prset ratepr = variable* (assigns a separate branch length parameter for each partition) was employed as per the recommendations of Marshall *et al.*[53], who found that BI analyses of partitioned data with a global branch length parameter resulted in significantly longer overall tree length. Four 5-million generation pilot analyses (*temp* = 0.2, 0.1, 0.02, 0.01) were run to determine the optimal temperature setting to assure an appropriate acceptance rate of swaps between chains [54]. Subsequently, two independent, simultaneous BI searches were run for 160 million generations, saving a tree every 5000 generations, with four search chains each (*temp* = 0.01). The analysis was terminated ~100 million generations after the average standard deviation of the split frequencies fell below 0.02. The 20,000 post-burn-in trees from each run, determined by examination of the log probability of observing the data by generation plot with Tracer (v. 1.5 [55]), were used to calculate the majority rule consensus tree whose nodal support values were plotted on the BI MAP tree (= maximum *a posteriori* probability tree).

A maximum likelihood (ML) tree was generated using GARLI (v. 2.0 [56]) using the same partitioning scheme and model assignments as the BI analysis (above) and using the default settings except for the following: *searchreps* = 5, *numberofprecreductions* = 20, *treerejectionthreshold* = 100. The parameter estimates from the search replicate that obtained the tree with the highest log-likelihood value were fixed in a 200-replicate ML bootstrap analysis [57] using default settings.

### Character optimizations

The estimation of ancestral feeding groups, based on the BI MAP tree and best ML tree, were carried out using ML methods in Mesquite (v. 2.74 [58]). We followed the behavioral groups of Knutson and Vala [17], which are based on the most recent analysis of sciomyzid life cycles. Our data set included taxa from ten sciomyzid behavioral groups plus *Pelidnoptera* (Phaeomyiidae) and the outgroup (*Drosophila*) as 11^th^ and 12^th^ states. The Markov k-state one parameter model (MK1 [59]) was used to infer ancestral character states in the ML optimizations. We also optimized larval habitat (coded as aquatic or terrestrial) for which we utilized the Asymmetrical Markov k-state 2 parameter model (AsymmMK; available only for binary characters [60-62]) which allows forward and backward rates to be different. This model was used because the behavioral group optimization estimated a 10:1 ratio of aquatic-to-terrestrial versus terrestrial-to-aquatic transitions. To make decisions regarding the significance of ancestral character state reconstructions, we followed Pagel [63] (following Edwards [64]) who recommended that ancestral character state estimates with a log-likelihood two or more units lower than the best state estimate (decision threshold [T] set to T = 2) be rejected. Generally viewed as a conservative cutoff, this threshold has been used by numerous recent authors (e.g., [65-69]). The DEC model implemented in the program Lagrange [32] was also used to estimate ancestral habitats using the BI MAP tree. Unlike the Mesquite ML optimization which assumes instantaneous habitat transitions, Lagrange models habitat evolution along branches (i.e., over time), therefore allowing ancestors to occur in two habitats simultaneously. While apparently rare in sciomyzids, we did have one taxon (*Tetanocera plumosa*; see *Hypothesis testing* section below) that is known to occur both in aquatic and shoreline habitats. Because the Mesquite optimization suggested a 10:1 ratio of aquatic-terrestrial vs. terrestrial-aquatic transitions, we optimized this parameter in Lagrange and used a 12:1 ratio to infer ancestral habitats.

### Hypothesis testing

Because many of Knutson and Vala's [17] behavioral groups within *Tetanocera* were not monophyletic in the unconstrained analyses, we conducted separate analyses constraining each of these groups to be monophyletic. Each resulting constrained tree was statistically compared (see below) to the unconstrained tree to test whether the monophyly of Knutson and Vala's [17] behavioral groups in *Tetanocera* could be rejected, thereby adding statistical support to inferences of multiple independent feeding behavior and habitat transitions. One unconstrained and six constrained analyses were done with RAxML (iMAC Pthreads-version [70,71]) using the same partitioning scheme as above under the GTR + G + I model [72]. Twenty replicate searches were done for each analysis (constrained and unconstrained) and the tree with the highest log-likelihood from each was used for topology testing (below). To assure that only topology changes within *Tetanocera* were the major differences between constrained and unconstrained trees, all but five outgroups were removed, leaving one individual each of *D. melanogasterAtrichomelina puberaAnticheta melanosomaHoplodictya acuticornis* and *Limnia boscii* and 54 *Tetanocera* terminals for which behavioral group is known (see Table 1). The non-*Tetanocera* taxa remaining in the analyses were chosen because each represents a major lineage in the BI MAP (maximum *a-posteriori* probability) tree and best ML trees and they had the lowest percentage of missing data. Behavioral groups that were not monophyletic on either the BI MAP or best ML trees were constrained in separate analyses as follows (see Table 1): *Aquatic1*: all *Tetanocera* with aquatic larvae including the facultative *T. plumosa* which can also occur on damp shorelines [73]; *Aquatic2*: same as *Aquatic1* excluding *T. plumosa*; *Shoreline1*: all *Tetanocera* with larvae occurring on damp shorelines and preying on aquatic snails excluding *T. plumosa*; *Shoreline2*: same as *Shoreline1* plus *T. plumosa*; *Slug*: all slug parasitoids; *Terrestrials*: both species predatory on terrestrial snails.

The full 115-taxon data set was used to test the monophyly of the Renocerinae, proposed by Verbeke [74] to include *Renocera* + *Anticheta*, two genera quite distantly separated in the BI and ML analyses presented herein. To constrain *Renocera* + *Anticheta* as a lineage outside of the other tribes, the Sciomyzini, Tetanocerini and *Renocera* + *Anticheta* were each constrained to be monophyletic with three separate constraint statements. Finally, this data set was also used to evaluate whether the BI MAP tree and the best ML tree were significantly different from one another.

To test for significant differences in topologies between unconstrained and constrained analyses, GARLI (v. 2.0), under the same partitioning scheme and models as the BI analysis, was used to create the site-likelihoods file used as input for the topology-testing program CONSEL (v. 0.1 k [75]). CONSEL was used to do the likelihood-based approximately unbiased test (AU [76]), Shimodaira-Hasegawa test (SH [77]), weighted Kishino-Hasegawa test and weighted Shimodaira-Hasegawa test (WKH and WSH [76]). Results of the KH test [78] were omitted due to its inappropriateness for testing *a posteriori* significant differences among tree topologies [79].

## Competing interests

The authors declare that they have no competing interests.

## Author’ contributions

Research was conducted by EGC, under the overall guidance of major professors WRH and BAF at Kent State University and postdoctoral advisor JDH at the University of Kentucky. All advisers were integral to the development, funding and execution of all aspects of research. AAP was involved in data collection of Palaearctic specimens and contributed to the writing of the manuscript.

## Supplementary Material

Additional file 1 **Figure S1.** Maximum likelihood tree produced by using the partitioning scheme and model assignments in Additional file 1: Table S2 using the default settings in Garli (v. 2.0) except for the following: *searchreps* = 5, *numberofprecreductions* = 20, *treerejectionthreshold* = 100. *Drosophila melanogaster* sequences were used to root the analysis. Numbers after species names are specimen numbers (Table 1). **Figure S2.** Bayesian consensus tree produced by using the partitioning scheme and model assignments in Additional file 1**: Table S2.***Drosophila melanogaster* sequences were used to root the analysis. Numbers after species names are specimen numbers (Table 1). **Figure S3.** Maximum likelihood bootstrap tree (200 replicates) produced by using the partitioning scheme and model assignments in Additional file **1****: Table S2** using the default settings in Garli (v. 2.0). Parameter estimates from the non-bootstrap search replicate that obtained the tree with the highest log-likelihood value were fixed. *Drosophila melanogaster* sequences were used to root the analysis. Numbers after species names are specimen numbers (Table 1). **Figure S4.** Maximum likelihood optimization of Knutson and Vala's [17] larval feeding groups on the maximum likelihood topology. Additional file 1: Figure S1; pruned to include only one terminal per species) analyzed with Mesquite using the MK1 model of character evolution. Only character states that are statistically significantly better than the others (ancestral character state estimates with a log likelihood two or more units higher than all others) are shown in the pie charts at the nodes. A solid (one color) node indicates that state is significantly better than all other possible states. Grey indicates unknown character states. Numbers after species names are specimen numbers (Table 1).** Table S1.** Model log-likelihood scores for variations of the ratio of aquatic-terrestrial:terrestrialaquatic transitions using the DEC model in Lagrange with dispersal and extinction rates for each. **Bold** font indicates significantly better log-likelihoods (i.e., greater than 2 lnL units) than the null (1:1) model. Red font indicates the parameter setting used in the plot of ancestral character states on Figure 3.** Figure S5.** Lagrange output with ratio of aquatic-to-terrestrial vs. terrestrial-to-aquatic transitions set to 12:1. Only states within 2 log-likelihood units of the best were considered for plotting on Figure 3, and only unambiguous states were plotted.** Table S2. **Gene information and evolutionary models selected by jModelTest for BI and ML phylogenetic analyses). Click here for file
