# Supplementary material for: Widespread and persistent invasions of terrestrial habitats coincident with larval feeding behavior transitions during snail-killing fly evolution (Diptera: Sciomyzidae)
Source: BMC Evol Biol. 2012 Sep 10;12:175. doi: 10.1186/1471-2148-12-175 (PMC3483186; doi:10.1186/1471-2148-12-175)
Supplement: Additional file 1 — Figure S1. Maximum likelihood tree produced by using the partitioning scheme and model assignments in Additional file 1: Table S2 using the default settings in Garli (v. 2.0) except for the following: searchreps = 5, numberofprecreductions = 20, treerejectionthreshold = 100. Drosophila melanogaster sequences were used to root the analysis. Numbers after species names are specimen numbers (Table 1). Figure S2. Bayesian consensus tree produced by using the partitioning scheme and model assignments in Additional file 1: Table S2.Drosophila melanogaster sequences were used to root the analysis. Numbers after species names are specimen numbers (Table 1). Figure S3. Maximum likelihood bootstrap tree (200 replicates) produced by using the partitioning scheme and model assignments in Additional file 1: Table S2 using the default settings in Garli (v. 2.0). Parameter estimates from the non-bootstrap search replicate that obtained the tree with the highest log-likelihood value were fixed. Drosophila melanogaster sequences were used to root the analysis. Numbers after species names are specimen numbers (Table 1). Figure S4. Maximum likelihood optimization of Knutson and Vala's [17] larval feeding groups on the maximum likelihood topology. Additional file 1: Figure S1; pruned to include only one terminal per species) analyzed with Mesquite using the MK1 model of character evolution. Only character states that are statistically significantly better than the others (ancestral character state estimates with a log likelihood two or more units higher than all others) are shown in the pie charts at the nodes. A solid (one color) node indicates that state is significantly better than all other possible states. Grey indicates unknown character states. Numbers after species names are specimen numbers (Table 1). Table S1. Model log-likelihood scores for variations of the ratio of aquatic-terrestrial:terrestrialaquatic transitions using the DEC model in Lagrange with dispersal and extinct [file 1471-2148-12-175-S1.pdf]

Table S1. Gene information and evolutionary models selected by jModelTest for BI and ML phylogenetic analyses.

Gene region	Genome: Gene Type	Position in data set	Evolutionary Model
COI 1st position	Mt: Protein-Coding	2-1474\3	HKY+I+G
COI 2nd position	Mt: Protein-Coding	3-1474\3	HKY+I+G
COI 3rd position	Mt: Protein-Coding	1-1474\3	K80+I+G
COII 1st position	Mt: Protein-Coding	1475-2155\3	GTR+G
COII 2nd position	Mt: Protein-Coding	1476-2155\3	GTR+G
COII 3rd position	Mt: Protein-Coding	1477-2155\3	HKY+G
16S	Mt: Ribosomal	3251-3676	HKY+I+G
28S	Nu: Ribosomal	2156-3250	GTR+I+G
Ef1α 1st position	Nu: Protein-Coding	3679-4552\3	HKY+G
Ef1α 2nd position	Nu: Protein-Coding	3677-4552\3	JC+I
Ef1α 3rd position	Nu: Protein-Coding	3678-4552\3	GTR+G
